# Equilibrium, Kinetics, and Thermodynamic Studies of Malachite Green Adsorption onto Fig (*Ficus cartia*) Leaves

**DOI:** 10.1155/2020/7384675

**Published:** 2020-03-04

**Authors:** Yemane Tadesse Gebreslassie

**Affiliations:** Department of Chemistry, Adigrat University, P.O. Box 50, Adigrat, Tigray, Ethiopia

## Abstract

The release of dyes from dying industries such as leather, paper, and textiles is an important cause of environmental pollution. In the present study, the batch adsorption measurements were carried out using stimulated aqueous solutions and the effect of operating variables such as initial malachite green concentration, amount of adsorbent, solution pH, contact time, and solution temperature, were investigated. The experimental result showed that the percentage removal decreased with an increase in initial dye concentration but increased as pH of the solution, contact time, and adsorbent dose increased. The equilibrium data were analyzed using Langmuir adsorption isotherm, Freundlich adsorption isotherm, and Tempkin isotherm models, and it was observed that the Langmuir adsorption isotherm better described the adsorption process. The monolayer adsorption capacity of activated carbon prepared from fig leaves for malachite green adsorption was found to be 51.79 mg/g at 298 K. Furthermore, the adsorption kinetics of the dye was investigated, and the rate of adsorption was found to follow the pseudo-first-order kinetic model with intraparticle diffusion as one of the rate-determining steps. The negative value of Δ*G*^0^ and the positive values of Δ*H*^0^ indicate the spontaneous and endothermic nature of the adsorption process, respectively. The experimental result obtained in the present study and comparison with other reported adsorbents indicate that activated carbon prepared from fig leaves could be used as a low-cost alternative adsorbent for the removal of malachite green from aqueous solution.

## 1. Introduction

Various manufacturing industries in the world, such as paper, pharmaceutical, leather, rubber, plastics, cosmetics, printing, and foods use dyes for coloring their end product. Among the various types of synthetic dyes, malachite green (MG) has been widely used for dyeing cotton, leather, wool, silk, paper, distilleries, jute, and so forth [1–3]. It is also used as a fungicide, antiseptic, and antiparasitical agent in aquaculture to control fish parasites and disease. But recently, its oral consumption by the animal has been found to be toxic, carcinogenic, mutagenic, and teratogenic [4–7]. Despite the fact that the use of this dye has been prohibited in several countries, it is still being used in many parts of the world due to its low cost, ready availability, and efficacy [5, 8]. The release of dyes into natural water bodies not only affect their aesthetic value but also interfere with sunlight penetration into the streams, reduces the solubility of dissolved oxygen, and therefore affects photosynthetic activity in aquatic life [9, 10]. Therefore, the safe disposal of dyes from industrial effluents is of great importance.

Several treatment methods including coagulation [11], chemical oxidation, photo-degradation [12], ultrasound-degradation [13, 14], reverse osmosis [15], biodegradation [16, 17], and adsorption [1–3, 6, 8, 10] have been reported for the removal of dyes from industrial effluents. However, some of these treatment methods have their own limitations such as high cost, high energy requirement, incomplete color removal, and generation of toxic waste products that need further treatment. Many dyes are stable to heat, light, and oxidizing agents which makes the conventional treatment difficult. [9, 18]. Adsorption has been found to be a cheap, eco-friendly, and biocompatible method for the removal of dyes from wastewater due to its ability to separate a wide range of compounds, abundant availability of low-cost adsorbents, simple design, and easy operation [6, 19]. Because of its high surface area, microporous structure, high adsorption capacity, and a significant degree of surface reactivity, commercial activated carbon is commonly used as an adsorbent for the removal of different types of dyes [19]. However, the extensive use of activated carbon is still challenging due to its high cost and complexity of the regeneration process [19, 20]. Recently, many researchers have been focusing on exploring cheaper and abundantly available adsorbents. Many low-cost adsorbents which are mainly from industrial and agricultural production, such as bagasse fly ash [1], *Pithophora* sp. [2], bottom ash [3], *Arundo donax* [6], oil palm trunk fiber [8], Sugarcane bagasse [9], neem leaves [20], *Prosopis cineraria* [21], *Pleurotus ostreatus* [22], *Limonia acidissima* [23], Rice husk [24], *Daucus carota* [25], sawdust [26], banana pseudo-stem fibers [27], ginger [28], *Solanum tuberosum* [29], rubber wood sawdust [30], rattan sawdust [31], and bamboo-based activated carbon [32] have been reported for the removal of MG from aqueous solution.

The objective of the present study was to use a chemically activated *Ficus cartia* leaves powder (AFLP) as an adsorbent for the removal of malachite green from aqueous solutions under different experimental conditions. The effect of experimental parameters such as the initial concentration of dye, amount of adsorbent, solution pH, temperature, and contact time was studied. Adsorption isotherm, kinetics, and thermodynamics of the adsorption of malachite green onto AFLP were also investigated.

## 2. Materials and Methods

### 2.1. Preparation of AFLP

Fig (*Ficus cartia*) leaves collected from a local area were washed repeatedly with distilled water to get rid of dust and other soluble impurities and dried at room temperature. The activation of the plant leaves was carried out by impregnation of dry fig leaves with 0.1 N phosphoric acids in a weight ratio of 1 : 2 (w/w) and heated in a muffle furnace at 200°C for 24 hours. The carbonized material was washed with distilled water and soaked in 2% NaHCO_3_ for 24 h to remove the remaining acid. After that, the product was dried in an oven at 105°C for 24 hours, grounded, and sieved through 200 mesh (<75 *μ*m) sieve to get AFLP. Finally, the sieved product was placed in a glass bottle and stored in a desiccator for further use.

### 2.2. Preparation of Stock Solution

To determine the applicability of AFLP as a potential adsorbent for wastewater treatment, malachite green (chemical formula = *C*_23_H_25_N_2_Cl, MW = 264.92  *λ*_max_=617 nm) was used as an adsorbate. A stock solution (1000 mg/L) of this dye was prepared by dissolving an accurately weighed amount of the dye in double distilled water. Experimental solutions of desired concentrations were prepared by successive dilutions of the stock solution using double distilled water. All the chemicals used were of analytical grade reagent.

### 2.3. Batch Adsorption Studies

In order to investigate the effects of the experimental parameters, such as solution pH, amount of adsorbent, initial dye concentration, contact time, and temperature, on the removal of MG by AFLP, batch adsorption experiments were performed by varying the parameters under investigation and keeping other parameters constant. In each experiment, a known amount of AFLP added to 100 mL of MG solution of known concentration and pH was taken in 250 mL conical flask. pH of the solutions was adjusted by adding a few drops of an aqueous solution of 0.1 M HCl or 0.1 M NaOH. Each flask was covered using aluminum foil and placed on a water bath shaker at a speed of 150 rpm for a given length of time at a predetermined temperature. After attaining equilibrium, the mixture in each flask was filtered using the Whatman filter paper and the residual MG concentration was determined using UV-vis spectrophotometer at  *λ*_max_=617 nm  (experimentally obtained). For better adsorption, the effect of solution pH on the removal of MG was examined over a pH range of 2 to 12. To optimize the amount of adsorbent, 100 mg/L of dye solution was contacted with adsorbent dose ranging from 50 to 500 mg. The effect of contact time was studied by varying time from 20 to 300 min and initial dye concentration ranging from 50 to 500 mg/L at a fixed adsorbent dose. The percentage removal and adsorption capacity, *q*_*e*_ (mg/g), of the adsorbent were calculated using equations (1) and (2), respectively [3]:

(1) $$\begin{array}{c} \text{Dye removal}\left(\%\right)=\frac{C_{0}-C_{e}}{C_{0}}\times 100\%, \end{array}$$

(2) $$\begin{array}{c} q_{e}=\left(C_{0}-C_{e}\right)\frac{V}{W}, \end{array}$$

where *C*_0_ and *C*_*e*_ are the initial and equilibrium concentrations of MG (mg/L), respectively; *V* is the volume of the dye solution (L), *W* is the weight of the adsorbent used (g), and *q*_*e*_ is the adsorption capacity per unit mass of adsorbent (mg/g).The adsorption capacity at a time, *t*, was determined using equation (3):

(3) $$\begin{array}{c} q_{t}=\left(C_{0}-C_{t}\right)\frac{V}{W}, \end{array}$$

where *C*_*t*_ is the concentration of MG at any time (mg/L). All adsorption experiments were performed in triplicate, and the mean values were used in data analysis.

## 3. Result and Discussion

### 3.1. Effect of Contact Time

**Figure 1:**
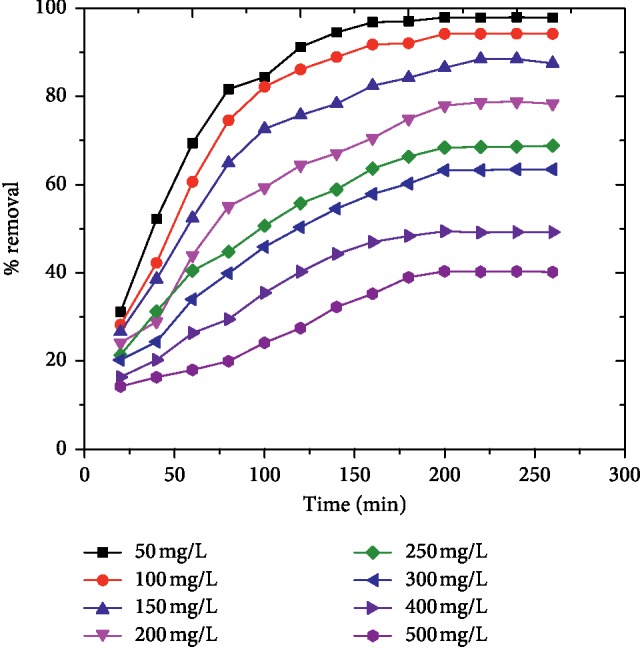
Effect of contact time on the adsorption of MG by AFLP (adsorbent dose = 400 mg, pH = 10, temp. = 298 K, and initial dye concentration: 50–500 mg/L).

The influence of contact time on the percentage removal of the dye by AFLP was investigated at different initial dye concentrations (50–500 mg/L) whilst maintaining adsorbent dose 400 mg and solution pH 10 at 298 K for different time intervals up to 300 min. As shown in Figure 1, the rate of adsorption rose rapidly at the beginning of the process and thereafter proceeds slowly with increasing contact times and finally reached equilibrium. The fast adsorption observed at the initial stage of the adsorption process is probably due to the copious availability of a negatively charged active site on the surface of the adsorbent. However, the slow rate of adsorption at later times is due to the electrostatic repulsion between the adsorbed MG cations and the cationic adsorbate species available in the solution. In addition, the slow pore diffusion of MG cations into the bulk phase of the adsorbent makes the adsorption process take a longer time to attain equilibrium. The equilibrium between the adsorbate and the adsorbent surface was attained at 200 min when the maximum MG adsorption onto the adsorbent was reached. The present findings were in good agreement with earlier reports [1, 6, 23, 26]. The smooth and continuous adsorption curves indicate the possible monolayer coverage of malachite green molecules on the surface of activated fig leaf powder [33].

### 3.2. Effect of Initial Dye Concentration

**Figure 2:**
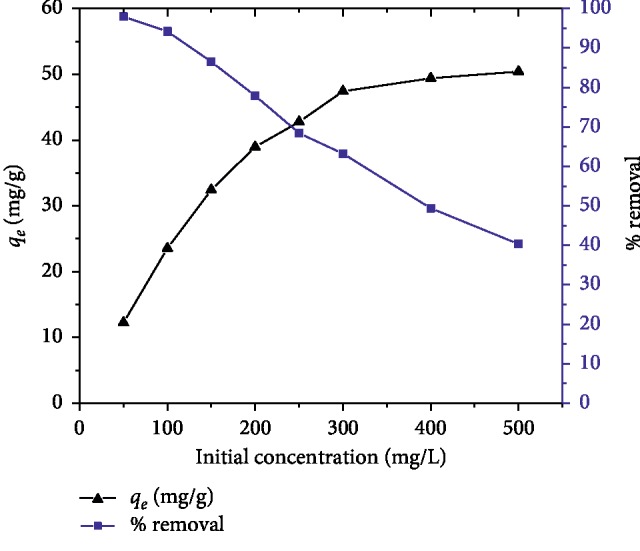
Effect of initial dye concentration on the adsorption of MG by AFLP (adsorbent dose = 400 mg/100 mL, temp. = 298 K time = 200 min, and pH = 10).

The effect of initial dye concentration on the adsorption process of MG was carried by varying the initial dye concentration from 50 to 500 mg/L while keeping other parameters constant (see Figure 2). It is evident from the figure that the percentage dye removal decreased from 97.96% to 40.34% for increasing initial MG concentration from 50 to 500 mg/L, but the actual amount of dye adsorbed per unit mass of adsorbent increased from 12.25 to 50.43 mg/L with increasing initial MG concentration. The higher dye percentage removal at a lower concentration is because the ratio of the number of dye molecules to the available active sites of the adsorbent is low, indicating a greater possibility of interaction between molecules of malachite green and the available active sites of the adsorbent. However, as the ratio of a number of molecules of dye to the number of surface active sites on the adsorbent increases, the active sites become saturated. No more active sites on the adsorbent would be available for the molecules of the dye, resulting in a decrease in the percentage removal of the dye.

### 3.3. Effect of Adsorbent Dosage

**Figure 3:**
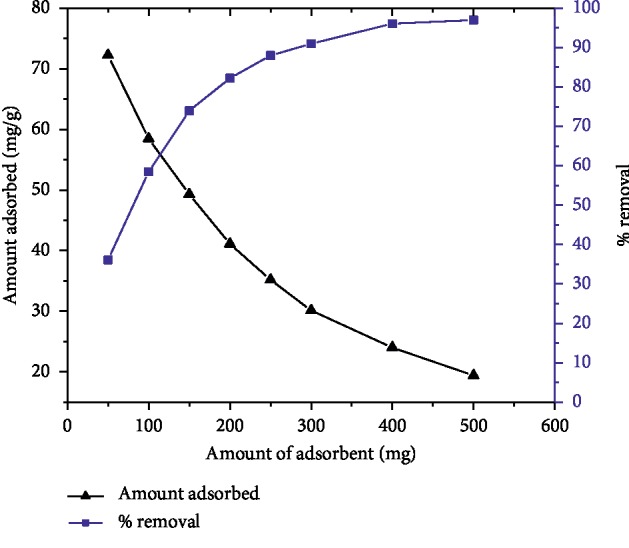
Effect of adsorbent dose on MG adsorption (*C*_0_ = 100 mg/L, pH = 10, temp. = 298 K, and contact time = 200 min).

The adsorption of MG on AFLP was studied by varying the amount of adsorbent from 50 to 500 mg while keeping the initial dye concentration at 100 mg/L, pH 10, the temperature at 298 K, and the equilibrium time at 200 min.  Figure 3 shows that the removal percentage of the dye increased from 36.12% to 96.96%, whereas the actual amount of dye adsorbed on the surface of the adsorbent decreased from 72.24 to 19.39 mg/g as the adsorbent dose increased from 50 to 500 mg. The enhancement in adsorption with dose is due to surface area increment and availability of more active sites on the adsorbent. As shown in Figure 3, a further rise in the amount of adsorbent to 500 mg did not show a significant increase in the percentage removal, confirming the dye is at equilibrium between the solid adsorbent and liquid phase. Therefore, 400 mg adsorbent dose was chosen for further experiments.

### 3.4. Effect of pH

**Figure 4:**
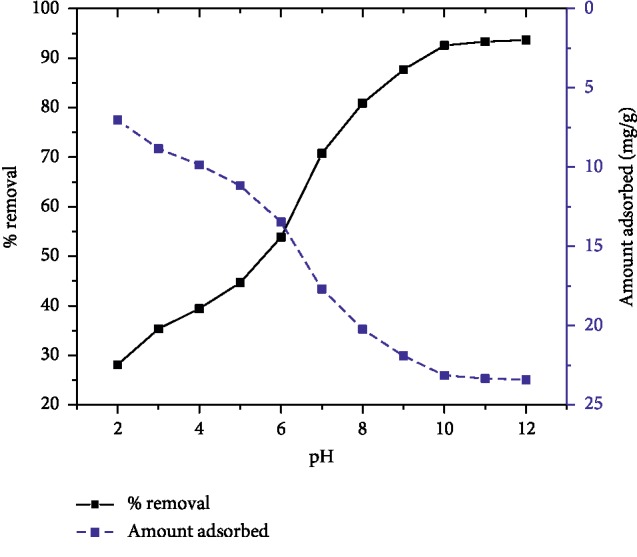
Effect of pH on MG adsorption (*C*_0_ = 100 mg/L, adsorbent dose = 400 mg/100 mL, temp. = 298 K, and contact time = 200 min).

The solution pH is one of the most important factors that determine the efficiency of adsorbent most likely because of its influence on the surface characteristics of the adsorbent and ionization of the adsorbate molecules. To study the effect of pH on the adsorption of malachite green onto AFLP, the experiment was carried out at 100 mg/L initial dye concentration with 400 mg adsorbent dose for equilibrium time of 200 min at 298 K temperature.  Figure 4 indicates as the solution pH increased from 2 to 12, the percentage removal of the dye and the amount of dye adsorbed increased from 24.12% to 94% and 7.03 mg/g to 23.41 mg/g, respectively. This indicates that the adsorption was significantly affected by the solution pH. At a lower pH, there are a large number of positively charged surface sites and less number of negatively charged active sites, which did not favor the adsorption of positively charged MG molecules because of the electrostatic repulsion between the positively charged adsorbent site and a cationic dye. Furthermore, the lower percentage removal of MG at acidic pH is due to the presence of excess H^+^ ions which compete with MG cations for the vacant adsorbent sites of AFLP. Quite the reverse, the surface of the adsorbent acquires more negative charge at higher pH which enhances the uptake of cationic dye due to electrostatic force of attraction. A similar observation was reported for the adsorption of MG on bagasse fly ash [1], *Prosopis cineraria* [21], *Limonia acidissima* [23], and sulfuric acid treated sawdust [26]. No significant change in the percentage removal of the dye was observed at higher pH values (pH > 10). Hence, further experiments were carried out at pH 10.

### 3.5. Adsorption Isotherms

**Figure 5:**
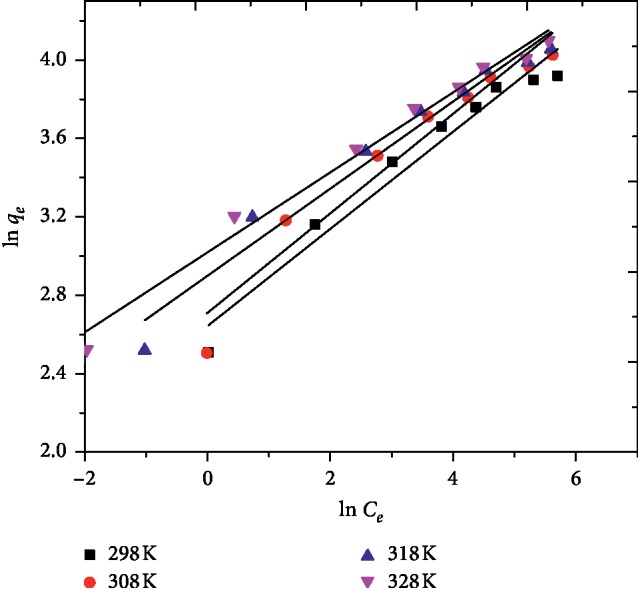
Freundlich isotherm for the adsorption of MG on AFLP (*C*_0_ = 100 mg/L, pH = 10, adsorbent dose = 400 mg/100 mL, and time = 200 min).

Adsorption isotherms are very important for understanding and describing the nature of interactions between the molecules of adsorbate and adsorbent centers. Different isotherms such as Freundlich, Langmuir, Dublin–Radushkevih (D-R), Redlich–Peterson (R–P), and Tempkin isotherms have been employed to describe the adsorption mechanism, the surface characteristics, and affinity of the adsorbent sites for the adsorbate. In this study Freundlich, Langmuir, and Tempkin isotherm models were used to analyze the experimental data. The Freundlich isotherm model is an earliest well-known empirical equation which suggests multilayer coverage and applies to adsorption on heterogeneous surfaces with the lateral interaction between adsorbed molecules. It also assumes that adsorption energy decreases exponentially on completion of the active centers of the adsorbent. In this study, the experimental data were fitted to the linear form of the Freundlich isotherm equation expressed as follows [34]:

(4) $$\begin{array}{c} \ln\;q_{e}=\;\ln\;K_{F}+\frac{1}{n}\ln\;c_{e}, \end{array}$$

where *q*_*e*_ is the amount of dye adsorbed at equilibrium (mg/g), *c*_*e*_ is equilibrium dye concentration in solution (mg/L), *K*_*F*_ and *n* are the Freundlich constants which are related to the adsorption capacity and adsorption intensity, respectively.  Figure 5 indicates a linear plot of ln *q*_*e*_ versus ln *c*_*e*_  for the adsorption of MG onto AFLP at different temperatures. The values of Freundlich constants (*K*_*F*_ and n) were calculated from the slope and intercept of the linear plot of equation (4) and are given in Table 1. These values show the increase of negative charge on the AFLP surface that raises the weak intermolecular forces like Van der Waals's forces between the adsorbent and dye molecules, which in turn increases the adsorption of the dye. The intensity of adsorption is an indication of the bond energies between the adsorbent and dye, and the possibility of slight chemisorptions rather than physisorption. The values of *n* were found in the range from 3.94 to 4.90 at different temperatures, indicating that adsorption of MG onto AFLP was favorable and there is a possibility of multilayer adsorption of the dye. This agrees well with the findings of other investigators [23].

**Figure 6:**
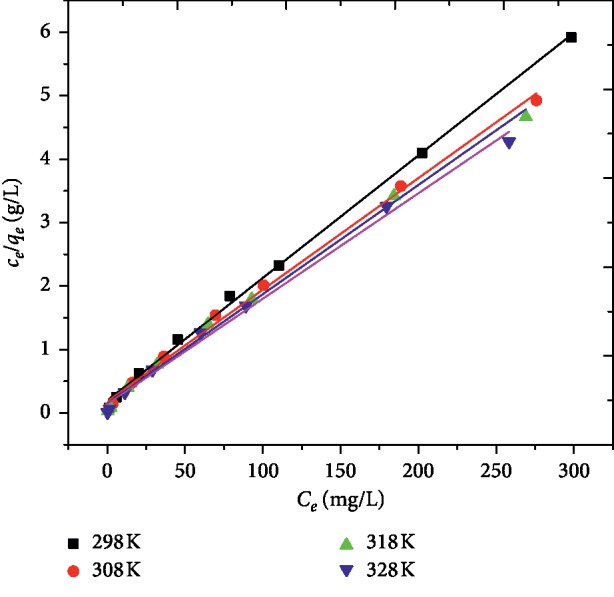
Langmuir isotherm for the adsorption of MG on AFLP (*C*_0_ = 100 mg/L, pH = 10, adsorbent dose = 400 mg/100 mL and time = 200 min).

Langmuir isotherm assumes monolayer adsorption of adsorbate from a solution on a homogeneous surface with a finite number of adsorption sites. Besides, the model assumes constant energy of adsorption without the transmigration of the adsorbate molecules in the plane of the surface. According to this model, there is a fixed number of adsorption sites on the surface of the adsorbent and once the active sites are completely occupied by a monolayer of the adsorbate, no further adsorption occurs [35, 36]. The Langmuir isotherm was employed for the determination of the maximum adsorption capacity corresponding to complete monolayer coverage on the adsorbent surface. The experimental data have been fitted to the linear form of Langmuir isotherm:

(5) $$\begin{array}{c} \frac{C_{e}}{q_{e}}=\frac{C_{e}}{q_{m}}+\;\frac{1}{K_{L}q_{m}}, \end{array}$$

where *q*_*m*_ is the monolayer adsorption capacity (mg/g) and *K*_*L*_ is Langmuir isotherm constant related to the affinity of the binding sites and energy of adsorption (L/mg). The values *q*_*m*_ and *K*_*L*_ were obtained from the slope and intercept of the linear plot of *C*_*e*_/*q*_*e*_ versus *C*_*e*_ (Figure 6) and are given in Table 1. The isotherm was found to be linear over the concentration range studied with good adjusted coefficient of determination (*R*^2^ > 0.994) for AFLP, showing the experimental data fitted well with the Langmuir model. Furthermore, analysis of the Langmuir adsorption isotherm model was made based on the dimensionless equilibrium parameter, *R*_*L*_, which is defined by [37]:

(6) $$\begin{array}{c} R_{L}=\frac{1}{1+K_{L}C_{0}}, \end{array}$$

where *C*_0_ is initial dye concentration (mg/L) and *K*_*L*_ is Langmuir isotherm constant (L/mg). It has been shown that the value of *R*_*L*_ indicates the type of Langmuir isotherm to be unfavorable (*R*_*L*_ > 1), favorable ( 0 < *R*_*L*_ < 1, *R*_*L*_ > 1), linear (*R*_*L*_=1) or irreversible (*R*_*L*_=0). The values of *R*_*L*_ for the adsorption process were found between zero and one, indicating the favorable adsorptive uptake of MG by AFLP [28, 31].

**Figure 7:**
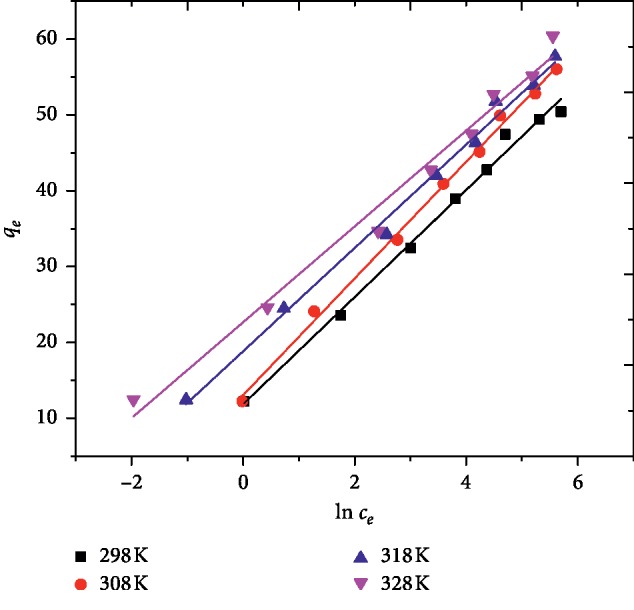
Tempkin isotherm for the adsorption of MG on AFLP (*C*_0_ = 100 mg/L, pH = 10, adsorbent dose = 400 mg/100 mL, and time = 200 min).

Tempkin isotherm model assumes the enthalpy of adsorption of all the molecules on the surface decreases linearly with coverage due to adsorbent-adsorbate interactions, and the adsorption is characterized by a uniform distribution of binding energies, up to maximum binding energy. For the Temkin model, the following linear equation was employed [38]:

(7) $$\begin{array}{c} q_{e}=B_{T}\ln\;K_{T}+\;B_{T}\ln\;C_{e}, \end{array}$$

where *B*_*T*_=*RT*/*b*, *R* is the universal gas constant, 8.314 J/mol·K, *T* is the absolute temperature in Kelvin, and *b* is Tempkin constant related to the heat of adsorption. *K*_*T*_ is the equilibrium binding constant corresponding to the maximum binding energy. The applicability of Tempkin isotherm was also investigated using the same set of experimental data, by plotting *q*_*e*_ versus ln *C*_*e*_ (Figure 7). The results obtained from the Tempkin isotherm model for the adsorption of MG onto AFLP and the corresponding adjusted coefficient of determination are shown in Table 1. The adjusted coefficient of determination, *R*^2^, obtained from this model was comparable with the Langmuir model, which proves the applicability of Tempkin isotherm for the adsorption of MG onto AFLP.

Further from Table 1, it was observed that the value of *R*^2^ was at a range between 0.937 and 0.982 for Freundlich isotherm, 0.994 and 0.998 for Langmuir isotherm, while for Tempkin isotherm was between 0.982 and 0.996. The highest *R*^2^ values for Langmuir isotherm model indicate an excellent fit with the experimental adsorption data at all temperatures. The fact that the experimental data fitted very well with Langmuir isotherm confirms the monolayer coverage of MG molecules onto AFLP surfaces as well as the uniform distribution of active sites on the surface of the adsorbent. It is also evident from Table 1 that the adsorption capacity, *q*_*m*_, and the adsorption energy, *k*_*L*_ of the adsorbent increases on raising the temperature, showing the endothermic nature of the adsorption process. From these results, we can conclude that the maximum adsorption corresponds to a saturated monolayer of MG molecules on the homogeneous active sites of the adsorbent surface and there was no transmigration of the MG molecules in the plane of the AFLP surface. The maximum adsorption capacity of the activated fig leaves powder was relatively large (51.79 mg/g) compared to some adsorbents reported in the literature, such as *Arundo donax* root carbon (8.69 mg/g) [6], banana pseudostem fiber (26.5 mg/g) [27], *Solanum tuberosum* (33.3 mg/g) [29], and rubber wood sawdust (36.45 mg/g) [30], revealing the potentiality of the AFLP as an adsorbent.

### 3.6. Kinetic Study of Adsorption

In order to examine the mechanism of adsorption of MG onto AFLP and determine the rate controlling steps like a chemical reaction, diffusion and mass transport process, three well-known kinetic models including pseudo-first order, pseudo-second order, and intraparticle diffusion model were applied to find the best-fitted model for the experimental data obtained. The pseudo-first order equation of Lagergren was applied to describe the adsorption of MG onto AFLP at different initial MG concentrations. The linear form of pseudo-first order rate equation is expressed as follows [6, 21, 23]:

(8) $$\begin{array}{c} \ln\left(q_{e}-q_{t}\right)=\;\ln\;q_{e}-k_{1}t, \end{array}$$

where *q*_*e*_ and *q*_*t*_ (mg/g) are the adsorption capacities at equilibrium and at a time *t*, respectively, and *k*_1_ (min^−1^) is the rate constant of pseudo-first order adsorption. The pseudo-second order kinetic model in linear form is expressed as follows [6, 23]:

(9) $$\begin{array}{c} \frac{t}{q_{t}}=\;\frac{1}{k_{2}q_{e}^{2}}+\;\frac{t}{q_{e}}, \end{array}$$

where *k*_2_ is the pseudo-second order rate constant (g/mg min).

**Figure 8:**
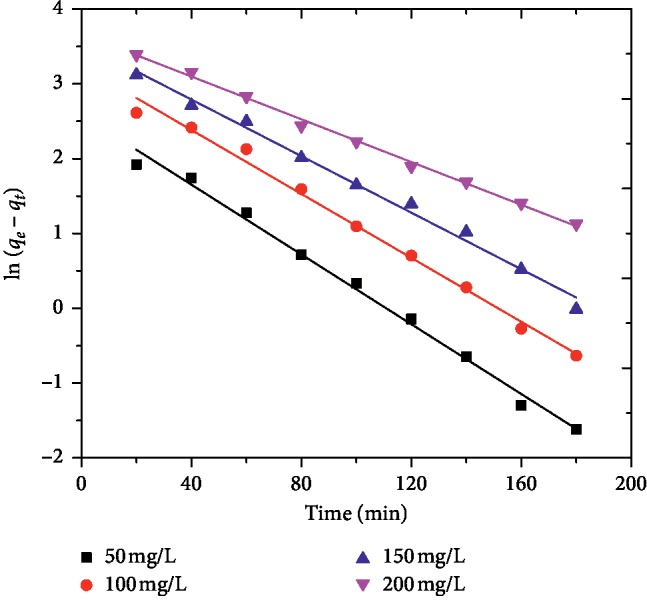
Pseudo-first order model for the adsorption of MG onto AFLP (pH = 0, dose = 400 mg/100 mL, time = 200, min and temp. = 298 K.

**Figure 9:**
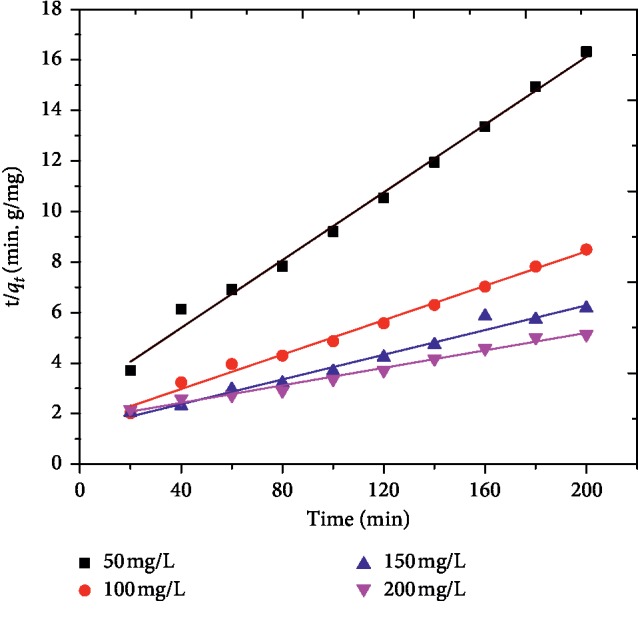
Pseudo-second order plot for the adsorption of MG onto AFLP (pH = 10, adsorbent dose = 400 mg/100 mL, time = 200 min, and temp. = 298 K.

By plotting ln(*q*_*e*_ − *q*_*t*_) versus *t* and *t*/*q*_*t*_ versus *t* for different initial MG concentrations, straight lines were obtained as shown in Figures 8 and 9. The kinetic constants calculated from the slope and intercepts of the plots of both models and their corresponding adjusted coefficient of determinations are given in Table 2. The results indicated that the *R*^2^ for both models were relatively high. However, the calculated *q*_*e*_, (*q*_*e*_, calc) values for the pseudo-first-order kinetic model were much closer to the experimental *q*_*e*,_ (*q*_*e*,_ exp) values than the *q*_*e*_ values for pseudo-second-order model. These findings suggest that the adsorption process of MG onto AFLP can be described by pseudo-first order kinetics. Similar adsorption kinetic results for MG by different adsorbents were reported by other researchers [21, 23, 26].

**Figure 10:**
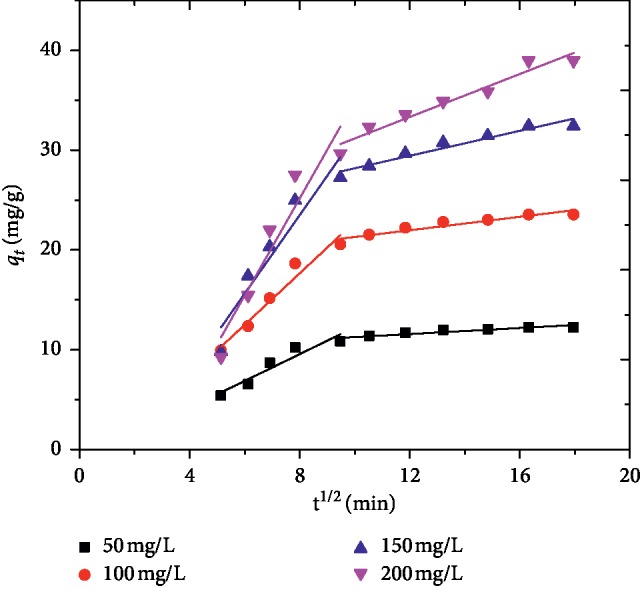
Intraparticle diffusion plot for the adsorption of MG onto AFLP (pH = 10, adsorbent dose = 400 mg/100 mL, time = 200 min).

Adsorption is a multistep process that involves the transport of the adsorbate molecules from the bulk of the solution into the solid phase followed by diffusion into the interior of the pores. Intraparticle diffusion rate equation was studied for the purpose of investigating the mechanism of the MG adsorption onto AFLP. The intraparticle diffusion model assumes that the adsorption capacity at a time *t* (*q*_*t*_) varies almost proportionally with *t*^1/2^ rather than with the contact time, *t* [39].

(10) $$\begin{array}{c} q_{t}=k_{\text{id}}t^{1/2}+\;C, \end{array}$$

where *C* is the intercept and *k*_id_ (mg/g min^1/2^) is the intraparticle diffusion rate constant, which can be calculated from the slope of the linear plot of *q*_*t*_ versus *t*^1/2^. The value of the intercept reflects the boundary layer effect and the larger the intercept value indicates the greater contribution of surface adsorption on the rate controlling step.  Figure 10 shows that there are two different regions in the curve indicating the different stages of the adsorption process, the initial portion is attributed to the bulk diffusion and the second portion to intraparticle diffusion [21].  Table 3 indicates that the intraparticle diffusion parameters for the adsorption of MG onto AFLP. The values of *R*^2^ were found to be in the range of 0.814 and 0.944, indicating that adsorption followed intraparticle diffusion. However, the lines did not pass through the origin. This indicates that intraparticle diffusion is not the sole rate controlling step, but there is also a contribution from the boundary layer effect. The values of C were increased with increased initial dye concentration, showing enhancement in thickness and the boundary layer. Comparable results were also reported by previous researchers [6, 21, 39–41].

### 3.7. Thermodynamic Study

**Figure 11:**
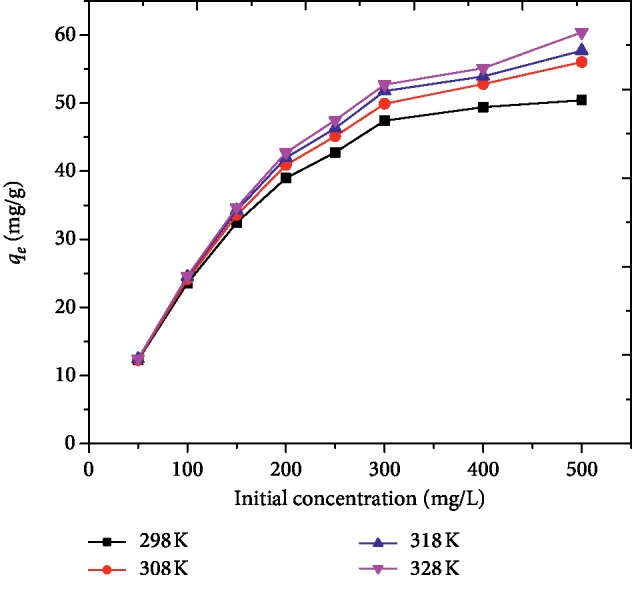
Effect of temperature on the adsorption of MG onto AFLP (pH = 10, adsorbent dose = 400 mg/100 mL, time = 200 min).

As shown in Figure 11, the influence of temperature on the adsorption of MG onto AFLP was studied by varying the temperature from 298 to 328 K. It was observed that as temperature increased the adsorption capacity of the adsorbent also increased, which revealed that the adsorption of MG on the adsorbent was endothermic in nature. A similar observation was also reported in the study of the adsorption of MG onto activated carbon from Pandanus leaves [42].

**Figure 12:**
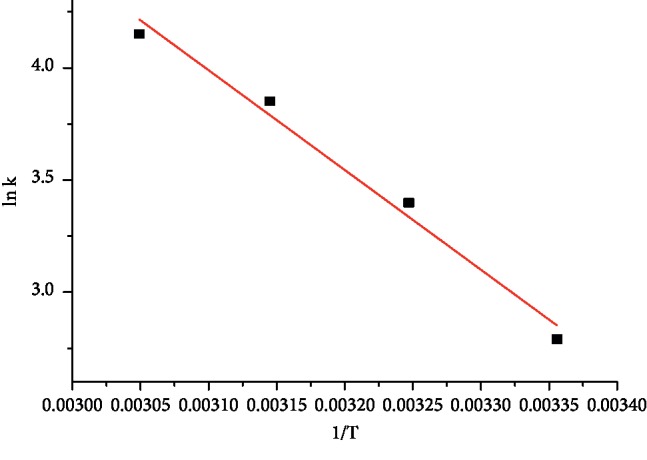
Plot of ln *K*_**c**_ versus T.

The thermodynamic parameters for the adsorption of MG onto AFLP, such as Gibbs energy (Δ*G*), enthalpy of adsorption (Δ*H*), and entropy of adsorption (Δ*S*) were examined using the following basic relations [28]:

(11) $$\begin{array}{c} \Delta G^{0}=-\text{RT}\ln\;K_{c}, \end{array}$$

(12) $$\begin{array}{c} K_{c}=\;\frac{C_{\text{ad}}}{C_{e}}, \end{array}$$

(13) $$\begin{array}{c} \ln\;K_{c}=\;\frac{\Delta S^{0\;}}{R}-\frac{\Delta H^{0}}{\text{RT }}, \end{array}$$

where *R* is gas constant (8.314 J/K.mol), *T* is temperature (K), *K*_*c*_ is equilibrium constant, *C*_ad_ and *C*_*e*_ corresponds to the equilibrium concentration on the adsorbent and solution, respectively. The values of Gibbs free energy change was calculated using equation (11) (Table 4). As can be depicted from Table 4, the negative value of Δ*G*^0^ shows the feasibility of the adsorption of MG on AFLP and the spontaneous nature of the adsorption process. The values of Δ*H*^0 ^ and Δ*S*^0^ were determined from the slope and intercept of the plot of ln *K*_*c*_ versus *T* (Figure 12) and are presented in Table 4. The positive value of Δ*H*^0 ^ shows that the adsorption is endothermic in nature and indicates the possibility of physisorption. The positive value of Δ*S*^0^ indicates the affinity of AFLP towards malachite green and increased disorder at the solid-solution interface during the adsorption process.

### 3.8. Comparison of Activated Fig Leaf Powder with Other Adsorbents

A comparative study of the maximum adsorption capacity of AFLP with those of other adsorbents employed for MG removal is given in Table 5. The adsorption capacity was low compared to some adsorbents such as rattan sawdust and bamboo-based activated carbon. However, such findings do not diminish the feasibility of using AFLP as an adsorbent for MG removal from aqueous solutions, because it presented good adsorption capacity in comparison to other low-cost adsorbents. The nature and properties of the adsorbents including surface area and the presence of different functional groups could be the main reason for the difference in the observed value of the adsorption capacity of various adsorbents. Compared to the previously reported adsorbents, fig leaves are abundantly available and considered as agricultural waste. Thus, AFLP can be utilized as an alternative adsorbent for the removal of MG from aqueous solutions.

## 4. Conclusion

In this study, the role of activated fig leaves powder for adsorption of MG from aqueous solution was investigated. The results of batch experiments for the removal of MG from aqueous solution showed that the percentage of dye removal increased with an increase in solution pH, adsorbent dose, contact time, and solution temperature. However, the percentage of removal has been found to decrease with an increase in initial dye concentration. The highest removal of MG was achieved using 400 mg/100 mL of adsorbent at a contact time of 200 min and pH 10. The adsorption data were best fitted to the Langmuir isotherm model suggesting monolayer coverage of the dye molecules on the surface of the adsorbent. The maximum adsorption capacity of AFLP was found to be 51.79 mg/g at 298 K. Adsorption kinetics of the adsorbent was well fitted to the pseudo-first-order equation. The thermodynamic parameters suggested that the MG adsorption onto AFLP was spontaneous and endothermic process. The result obtained in this study demonstrates that activated fig leaves powder can be used as an inexpensive and abundantly available alternative adsorbent for MG removal from aqueous solutions.

## Figures and Tables

**Table 1:** Isotherm parameters obtained by using the linear method for the adsorption of MG using AFLP at different temperatures.

Isotherm	Parameters	Temperature (K)			
		298	308	318	328
Freundlich	*K*_*f*_	11.74	15.03	18.16	20.47
	*n*	4.04	3.94	4.50	4.90
	*R* ^2^	0.954	0.937	0.959	0.982

Langmuir	*q*_*m*_ (mg/g)	51.79	56.91	58.12	60.20
	*K*_*L*_ × 10^−3^ (L/mg)	1.89	1.65	1.96	1.97
	*R*_*L*_	0.914–0.514	0.924–0.548	0.911–0.505	0.910–0.504
	*R* ^2^	0.998	0.996	0.996	0.994

Tempkin	*B*_*T*_ (mg^−1^)	7.061	7.694	6.814	6.317
	B	350.87	332.80	387.99	431.73
	*K*_*T*_ (L^−1^)	5.35	5.45	15.84	36.12
	*R* ^2^	0.991	0.996	0.992	0.982

**Table 2:** Kinetic parameters for the adsorption of MG onto FLP at 298 K and different initial concentrations.

*C*_0_ (mg/L)	*q*_*e*_, exp (mg/g)	Pseudo-first order			Pseudo-second order		
		*k*_1_ (min^−1^)	*q*_*e*_, cal (mg/g)	*R* ^2^	*q*_*e*_, cal (mg/g)	*k*_2_ × 10^−3^ (g/mg min)	*R* ^2^
50	12.25	0.0234	13.27	0.992	14.92	1.656	0.993
100	23.55	0.0214	25.50	0.991	29.36	0.720	0.991
150	32.44	0.0189	34.69	0.991	40.92	0.427	0.976
200	38.97	0.0143	39.24	0.993	57.77	0.173	0.985

**Table 3:** Parameters for intraparticle diffusion model on the adsorption of MG by AFLP at different initial dye concentration.

*C*_0_ (mg/L)	*k*_id_ (mg/g min^1/2^)	C (mg/L)	*R* ^2^
50	0.154	9.74	0.814
100	0.332	17.92	0.871
150	0.625	21.93	0.926
200	1.073	20.40	0.944

**Table 4:** Thermodynamic parameters for the adsorption of MG onto AFLP.

−Δ*G*^0^ (kJ/mol)				Δ*H*^0^ (kJ/mol)	Δ*S*^0^ (J/k.mol)
298 K	308 K	318K	328 K		
9.592	9.667	13.024	16.021	36.969	147.77+

**Table 5:** Comparison of adsorption capacity and pseudo-first order rate constant of AFLP and other adsorbents on the adsorption of malachite green.

Adsorbent	Condition		*q*_*m*_, mg/g	*k*_1_ (min^−1^)	References
	Temp., K	pH			
*Arundo donax* root carbon	303	5–7	8.6	0.026	[22]
Banana pseudo-stem fiber	298	7	26.5		[27]
Kapok hull activated carbon	300	6.7	30.16	0.006	[43]
Leaves of *Solanum tuberosum*	303	7	33.3	0.17	[29]
Wood apple shell	299	7.5	34.56		[23]
Rubber wood sawdust	305	—	36.45		[30]
Activated carbon of fig leaves	298	10	51.79		This study
Rattan sawdust	303	9–12	62.7		[31]
Treated ginger waste	303	9	84		[28]
Bentonite	298		178.6		[43, 44]
Bamboo-based activated carbon	303		263.6		[33]

## Data Availability

The data used to support the findings of this study are included within the manuscript. The author is also ready to provide all the necessary information about the article.
